# The Hypoglycaemic Effects of the New Zealand Pine Bark Extract on Sucrose Uptake and Glycaemic Responses in Healthy Adults—A Single-Blind, Randomised, Placebo-Controlled, Crossover Trial

**DOI:** 10.3390/nu17142277

**Published:** 2025-07-09

**Authors:** Wen Xin Janice Lim, Rachel A. Page, Cheryl S. Gammon, Paul J. Moughan

**Affiliations:** 1 Riddet Institute, Massey University, Palmerston North 4442, New Zealand; p.j.moughan@massey.ac.nz; 2 School of Health Sciences, Massey University, Wellington 6021, New Zealand; r.a.page@massey.ac.nz; 3 School of Health Sciences, Massey University, Auckland 0632, New Zealand; c.gammon@massey.ac.nz

**Keywords:** bioactives, hyperglycaemia, glucose intolerance, plant extract, postprandial glucose, postprandial insulin, proanthocyanin, sucrose inhibition, type 2 diabetes

## Abstract

Background: The New Zealand pine bark has been demonstrated in vitro to inhibit digestive enzymes involved in carbohydrate digestion (alpha-amylase, alpha-glucosidase, and dipeptidyl-peptidase 4 (DPP-4)). Objective: This study aims to investigate the inhibitory effects of the New Zealand pine bark on sucrose uptake and glycaemic responses in humans. Methods: A single-blind, randomised, placebo-controlled, crossover trial was carried out involving healthy adults (n = 40 (M: 12, F: 28), 30.1 ± 1.3 years, BMI 23.4 ± 0.5 kg/m^2^, HbA1c 32.5 ± 0.6 mmol/mol, FBG 4.7 ± 0.1 mmol/L). A control (75 g of sucrose powder only), and two doses of the pine bark extract (50 and 400 mg) were provided on separate occasions, with 75 g of sucrose mixed in 250 mL of water. Blood samples were collected at −10, 0, 15, 30, 45, 60, 90, and 120 min via a finger prick test. A linear mixed model for repeated measures (SPSS v30, IBM) was applied, and data presented as model-adjusted mean ± SEM. Results: Compared to control (247.5 ± 14.0 mmol/L⋅min), the iAUCglucose was significantly reduced with the 400 mg dose (211.8 ± 13.9 mmol/L⋅min, 14.4% reduction, and *p* = 0.037), but not with 50 mg dose (220.8 ± 14.2 mmol/L⋅min, 10.8% reduction, and *p* = 0.184). Compared to control (9.1 ± 0.2 mmol/L), glucose peak value was significantly reduced with the 50 mg dose (8.6 ± 0.2 mmol/L, 5.5% reduction, and *p* = 0.016) but not with the 400 mg dose (8.7 ± 0.2 mmol/L, 4.4% reduction, and *p* = 0.093). There were no statistically significant changes in postprandial insulin levels with the pine bark extract compared to control. Conclusions: The New Zealand pine bark extract attenuated sucrose uptake with improved glycaemic responses, and may therefore be useful as a hypoglycaemic adjunct to the diet.

## 1. Introduction

Worldwide, there is currently an increasing prevalence of prediabetes (impaired glucose tolerance, IGT) and similarly for diabetes [1]. Presently, 589 million adults (20–79 years), equivalent to 1 in 9, are living with diabetes, and this is predicted to rise to 853 million by 2050 (45% increase) [1]. Approximately 75% of all foods and beverages contain added sugars in various forms [2]. Habitual overconsumption of dietary sugars, such as glucose and sucrose (which is the predominant sugar in the modern diet), and starches increases energy intake and this coupled with a sedentary lifestyle contributes to obesity risk and the development of cardiometabolic diseases such as type 2 diabetes (T2D) [2,3].

A range of plant-derived, antioxidant-rich extracts including pine bark extracts has been shown to improve glycaemia in both healthy individuals and those with impaired glycaemic control or T2D [4,5,6,7,8]. As such, these extracts including pine bark may have potential to improve glycaemic control within at-risk populations, either as a dietary adjunct or incorporated into a functional food product.

The New Zealand pine bark extract is obtained from 15–30 year old Pinus radiata trees grown in New Zealand [9]. The Pinus radiata belongs to the family Pinaceae which is a fast-growing evergreen conifer [9,10]. The pine bark undergoes an extensive water-based extraction process that involves grinding, washing, extraction with deionized hot water (95–99 °C for 30 min), the removal of extracted solids, the cooling of raw liquor by heat exchanger, the concentration of raw liquor by reverse osmosis to approximately 25% dissolved solids whilst continuously removing undissolved solids, freeze drying, further grinding, and lastly blending [9]. The pine bark extract then undergoes a quality control check, where physical, chemical, microbiological, heavy metal, herbicide, and pesticide analyses are carried out to ensure consistent conformity with its product specifications [9]. The dry powder contains greater than 80% proanthocyanidins, 1–2% taxifolin, other flavonoids and phenolic acids (approximately 8%), and some carbohydrates (5–10%) [9]. The extract reportedly contains a higher concentration of proanthocyanidins than the French Maritime pine bark (Pycnogenol^®^), which contains 65–75% total procyanidins [11]. The New Zealand pine bark extract has been shown to reduce postprandial glucose in normoglycaemic, healthy participants at doses of 50 and 400 mg [7]. A recent in vitro study on the New Zealand pine bark extract has further provided supporting evidence of its glucose-lowering effects by its ability to inhibit alpha-amylase, alpha-glucosidase, and dipeptidyl-peptidase 4 (DPP-4) enzymes [12]. The enzymatic inhibition attributes of the pine bark extract may play a relevant role in reducing or slowing postprandial glucose absorption [13,14,15], or enhancing the incretin effect [16,17], potentially improving glycaemic responses in humans.

The hypoglycaemic effect of New Zealand pine bark may result from its high concentration of oligomeric proanthocyanidins in varying degrees of polymerisation [9], and unique structural properties such as hydrogen moieties (e.g., OH) and double bonds that determine its effective interaction with digestive enzymes, trans-membrane glycoproteins such as DPP-4, and apically located transporters and receptors involved in glucose absorption [11,18,19].

The present study investigates if the New Zealand pine bark is able to reduce postprandial glycaemia with sucrose as the carbohydrate load instead of glucose. We hypothesise that the New Zealand pine bark has the potential to inhibit the enzyme sucrase leading to a delayed or reduced digestion of sucrose (table sugar, also known as disaccharide composing glucose and fructose linked by an alpha-1,2-glycosidic bond), thereby improving postprandial glycaemic responses. This hypothesis is in line with the action of Acarbose, a known alpha-glucosidase inhibitor (antidiabetic drug), and other plant extracts, such as mulberry leaf extract, which have demonstrated inhibitory actions on enzymes that digest various types of carbohydrates including maltose, maltodextrin, and sucrose [20,21,22]. This suggests that the New Zealand pine bark may influence glucose absorption from sucrose by both reducing glucose absorption [7] and/or inhibiting sucrose digestion, thus improving the overall glycaemic responses.

To date there has been no study examining the effects of the New Zealand pine bark on sucrose and its impact on postprandial glucose and insulin responses in healthy humans. This study aimed to determine if the New Zealand pine bark extract of two doses (50 and 400 mg) were able to improve glycaemic and insulinaemic postprandial responses in healthy participants when co-administered with 75 g of sucrose solution.

## 2. Materials and Methods

### 2.1. Study Population

This study was approved in March 2024 by the Central Health and Disability Ethics Committee (HDEC) (2024 EXP 19678). The clinical trial was registered prospectively at anzctr.org.au (Australia New Zealand Clinical Trials Registry Number: ACTRN12624000491561). This study was conducted in accordance with the Declaration of Helsinki and all participants gave their informed consent prior to participating in the study.

Participants were recruited from Auckland, New Zealand using poster advertisements within the local university and community (May–November 2024). They were selected according to the following inclusion criteria: (i) Body Mass Index (BMI) of 18.5–29.9 kg/m^2^, (ii) aged 18–60 years, (iii) not suffering from any impaired glycaemic control ((fasting blood glucose (FBG) < 5.6 mmol/L) and glycated haemoglobin A1c (HbA1c) < 40 mmol/mol (Cobas b 101 HbA1c test, CV 0.8–1.7%, Roche Diagnostics)), (iv) not taking any forms of glucose-lowering medications or medications that may affect glucose metabolism, and (v) free from any form of illnesses or chronic diseases. Participants were excluded from the study if they had any form of cardiovascular or metabolic diseases, digestive ailments, if they smoked, were pregnant or lactating, and if they had any known allergies to pine bark. Participants were also required to complete the International Physical Activity Questionnaire (IPAQ) and The Eating Attitudes Testing (EAT-26) to determine the general physical activity level of the participants, and to ensure they did not have any eating disorders that might confound with the study data collected.

### 2.2. Study Design

This was an acute, randomised, single-blind, crossover, placebo-controlled study conducted at the nutrition research facility at Massey University, Auckland, New Zealand. The order of intervention of treatment assigned to each participant was randomised using a computer-generated random allocation sequence.

Participants attended the study visits in the morning in a fasted condition (no food for at least 12 h, except water). A baseline sample was taken at 10 min prior to the start of intervention. The participants were then told to consume the treatment within five minutes. Further blood samples were taken at time points 15, 30, 45, 60, 90, and 120 min. Blood samples were obtained via the finger prick test.

Glucose readings were obtained using a single-use disposable lancet (Accu-Chek Safe T-Pro Plus, Roche Diagnostics, Indianapolis, USA) and glucose meter (MediSense, Optium, Abbott, Auckland, New Zealand, 2.7–4.0% CV). Blood samples (300 μL) were collected into microvette capillary blood collection tubes (Sarstedt Microvette CB300 Lithium Heparin, Nümbrecht, Germany) treated with heparin for plasma insulin analysis. The samples were centrifuged at 2000× *g* for 5 min according to the manufacturer’s instructions to obtain 200 μL supernatant, which was then stored in 200 μL aliquot and kept in −80 °C freezer until the plasma insulin analysis. Plasma insulin was measured by a two-site sandwich immunoassay using direct chemiluminescent technology (Atellica IM Analyzer, CV 1.4–1.8%, Siemens Healthcare Limited, Auckland, New Zealand) [23].

At each study visit, all participants were checked for dietary and lifestyle compliance, such as having fasted at least 12 h prior to each study visit, having a consistent diet, refraining from strenuous physical activity, alcohol, caffeinated tea or coffee formulations, health supplements 24 h prior to visit, any supplements or medications that may influence glucose levels, and no consumption of pine bark throughout the duration of the study. Research has demonstrated that pine bark extract undergoes extensive metabolism and is typically eliminated from the human body within 24 h [11]. Therefore, a washout period consisting of at least a 48–72 h gap to a week was observed between each study visit to minimise carry-over effects.

### 2.3. Intervention Treatments

Three intervention treatments were given to each participant at separate visits: control using a placebo (75 g of sucrose powder only), and two doses of the New Zealand pine bark extract (50 and 400 mg). Each intervention extract was prepared with 75 g of sucrose powder dissolved in 250 mL of water with 3.75 mL of artificial lemon flavour, and served to participants in an opaque, black cup (to conceal contents). All intervention treatments were of similar taste, flavour, and consistency to mask the contents given. Each intervention treatment was prepared fresh on the day of the study visit.

The New Zealand pine bark extract doses (50 and 400 mg) chosen for this study were based on our previous work on glucose metabolism in healthy participants demonstrating that both doses were effective in improving acute glycaemic control [7]. This was supported by other studies investigating the impact of pine bark (French Maritime) using doses ranging from 50 to 300 mg on longer-term (3 to 12 weeks) glycaemic control in people with T2D [24,25,26]. The pine bark extract has been vigorously tested for its safety for human consumption. Two human studies involving the consumption of 480 mg/day of the pine bark extract for 6 months and 960 mg/day for 5 weeks demonstrated no adverse effects on liver and kidney function, and haematology in the participants tested [9].The doses selected represented concentrations with no known toxicity [9]. The New Zealand pine bark extract (Enzogenol^®^) was supplied by ENZO Nutraceutical Limited (Paeroa, New Zealand), and was produced to the standards of dietary supplement preparations and quality control in New Zealand [9].

### 2.4. Sample Size

Following a similar study protocol that was used for the study examining mulberry leaf extract [22], it was estimated that n = 34 participants in each treatment group were required to detect a statistically significant difference (*p* ≤ 0.05) in incremental area under the curve (iAUC) between treatments of 43.1 mmol/L/min with a SD of 53.02 mmol/L/min. A total of n = 44 participants were recruited to factor in recruitment attrition and dropouts.

### 2.5. Statistical Analysis

Primary outcomes such as fasting and postprandial plasma glucose and insulin concentrations were measured at time points −10, 0, 15, 30, 45, 60, 90, and 120 min. The iAUC of both glucose and insulin were geometrically calculated using the trapezoidal rule and compared amongst the intervention treatments [27,28]. Secondary measures including the peak glucose, peak insulin, 1 h glucose, 1 h insulin, time of peak glucose, and time of peak insulin were calculated, similar to the study parameters measured in the mulberry leaf extract study [22]. IBM SPSS statistics version 30 (IBM corporation, New York, NY, USA) was used for statistical analysis. A linear mixed model for repeated-measures was performed suitable for a crossover study that also accounted for missing data values, and a Bonferroni post-hoc test for pairwise comparisons. Prior to statistical analysis, data normality was tested using the Kolmogorov–Smirnov and Shapiro–Wilks statistics. A *p* value of ≤0.05 was considered to be significant (95% confidence level), based on 80% power with alpha = 0.05 and beta = 0.10. The data were expressed as model-adjusted mean ± standard error of the mean (SEM). 

## 3. Results

### 3.1. Participant Demographics

**Figure 1:**
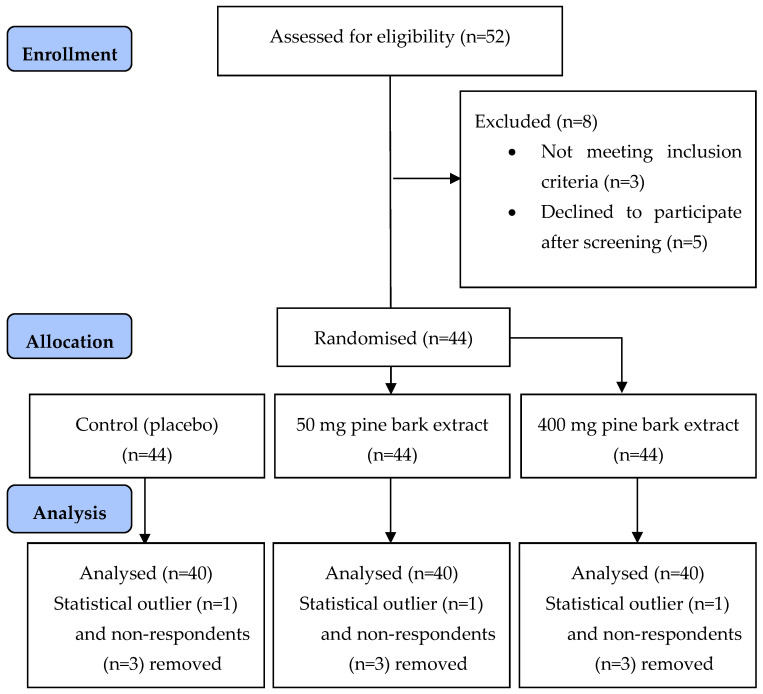
CONSORT diagram for the pine bark Study.

A total of 44 participants were recruited for the study (Figure 1), and 40 participants (M: 12, F: 28) completed the study. Data from participants who did not complete or withdrew from the study were included in the data analysis. One participant was detected to be an outlier (significantly elevated iAUCglucose values compared to other participants) and was removed from the data analysis. There were three participants who were non-respondents in the study (with iAUCglucose of control significantly lower than other participants and compared to all treatments tested) and they were also removed from the data analysis. The removal of the outlier and non-respondents improved the normality of the data. The participants who completed the study and data from withdrawn participants were included in the data analysis were healthy adults (n = 40 (M: 12, F: 28), 30.1 ± 1.3 years, BMI 23.4 ± 0.5 kg/m^2^, HbA1c 32.5 ± 0.6 mmol/mol, FBG 4.7 ± 0.1 mmol/L), and their blood pressure and lipid profile were within normal ranges (Table 1). The ethnicity distribution of the participants was 62% Asian (total n = 25, 24% South Asian (n = 6), 36% Southeast Asian (n = 9), 40% East Asian (n = 10)), 27% Caucasian (n = 11), 8% Middle Eastern (n = 3), and 3% African (East African (n = 1)). The IPAQ obtained at screening indicated that n = 3 (7%) participants had a low physical activity level, n = 19 (48%) had a moderate level of physical activity level, while n = 18 (45%) participants had a high physical activity level. The EAT-26 questionnaire given at screening did not detect any propensity for any eating disorder in participants recruited for the study. There were no adverse events reported during the course of the study.

### 3.2. Changes in Postprandial Glucose

**Figure 2:**
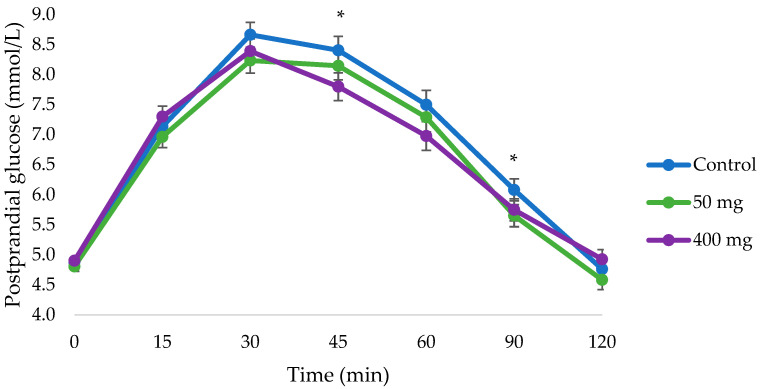
Changes in mean postprandial glucose (mmol/L) (± SEM) from 0 to 120 min of participants (n = 40) in each intervention (50 and 400 mg of pine bark extract compared to control). * Statistically significant differences were detected at 45 min with 400 mg of pine bark extract and 90 min with 50 mg of pine bark extract compared to control (*p* < 0.05).

Compared to control (247.5 ± 14.0 mmol/L⋅min), the iAUCglucose was significantly reduced with 400 mg of pine bark extract (211.8 ± 13.9 mmol/L⋅min, 14.4% reduction, and *p* = 0.037), but no significant change was detected with 50 mg of pine bark extract (220.8 ± 14.2 mmol/L⋅min, 10.8% reduction, and *p* = 0.184). Referring to Table 2, the significant reduction in iAUCglucose with 400 mg of pine bark extract could be seen at 90 min (*p* = 0.029) and 120 min (*p* = 0.037). Compared to the control (9.1 ± 0.2 mmol/L), there was also a significant reduction in the glucose peak value with 50 mg but not 400 mg of pine bark extract (8.6 ± 0.2 mmol/L, 5.5% reduction, *p* = 0.016, and 8.7 ± 0.2 mmol/L, 4.4% reduction, *p* = 0.093, respectively) (Table 2). Postprandial glucose was also significantly lower at 45 min with 400 mg of pine bark extract (*p* = 0.010), and at 90 min with 50 mg of pine bark extract (*p* = 0.023) compared to the control (Table 3 and Figure 2). There was only a statistical dose differences in the glucose peak time between 50 mg (36.7 ± 1.9 min) and 400 mg (31.3 ± 1.8 min) of the pine bark extract, *p* = 0.05 (Table 2).

### 3.3. Changes in Postprandial Insulin

**Figure 3:**
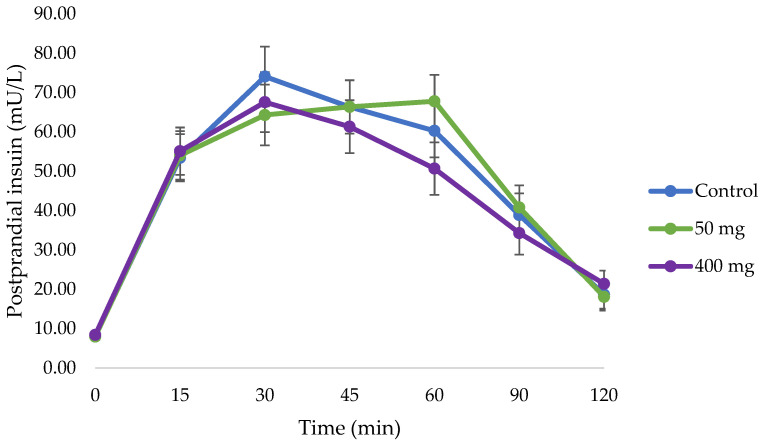
Changes in mean postprandial insulin (mU/L) (±SEM) from 0 to 120 min of participants (n = 40) in each intervention (50 and 400 mg of pine bark extract compared to control).

There were no statistically significant changes detected with either 50 mg of pine bark extract (4874.0 ± 504.5 mU/L⋅min, *p* = 1.000) or 400 mg of pine bark extract (4231.9 ± 498.3 mU/L⋅min, *p* = 0.949) compared to the control (4601.9 ± 499.4 mU/L⋅min) (Table 4 and Figure 3). There were also no statistically significant changes in 1 h insulin, peak insulin, and insulin peak time with the pine bark treatments compared to control, *p* > 0.05 (Table 4 and Table 5). There was only a significant dose difference in 1 h insulin concentrations between 50 and 400 mg of the extract (67.77 ± 6.70 mU/L vs. 50.66 ± 6.69 mU/L, respectively, *p* = 0.019).

## 4. Discussion

The New Zealand pine bark (*Pinus radiata*) extract and other similar pine bark extracts have been extensively studied for their hypoglycaemic effects in both healthy humans and individuals with T2D [9,10,24,25,26,29,30]. To the best of our knowledge, this is the first study that examines the effect of the New Zealand pine bark on sucrose digestion in improving glycaemic responses in human subjects. The current findings demonstrate that a higher dose of the pine bark extract (400 mg) significantly reduced overall mean iAUC of blood glucose with sucrose consumption. The pine bark extract was postulated to inhibit the sucrase enzyme leading to an attenuation in sucrose digestion. Equally, the pine bark extract may have attenuated glucose absorption. As the New Zealand pine bark extract mainly comprises large molecular structures of proanthocyanidins with varying degrees of polymerisation typically from 3 to 11, it was potentially able to inhibit both the digestive enzymes and glucose transporters in the small intestine via steric hindrance thus hindering the digestion of carbohydrates and glucose absorption [11,18,19], as observed in the current study. Even though 50 mg of pine bark dose showed no statistically significant reduction in iAUCglucose, it did significantly reduce the glucose peak value compared to the control (*p* = 0.016), and thus may be associated with potential reduction in oxidative stress and cardiometabolic (T2D and cardiovascular disease) risk [31,32]. Although the higher dose of pine bark (400 mg) tended to have a lower glucose peak compared to the control (*p* = 0.093), it did not reach statistical significance. An explanation might be, that at a 400 mg dose, there was a significantly delayed attenuation of overall postprandial glucose, notably only from 90 min onwards (Table 2), which was later than when the glucose peaked at approximately 30 min. However, 400 mg of pine bark extract had an earlier glucose peak time (31.3 ± 1.8 min) compared to the 50 mg dose (36.7 ± 1.9 min) and the control (35.8 ± 1.8 min), where the glucose peak time of 400 mg dose was significantly shorter than the 50 mg dose (*p* = 0.05). A shorter time to glucose peak has been associated with better glycaemic control and beta-cell function, and reduced T2D risk [33].

Whilst there was a statistically significant reduction in postprandial glucose with 400 mg of pine bark extract, the blood glucose reductions or pine bark concentration given did not appear to have been sufficient to elicit a change in overall postprandial insulin levels or to down-regulate insulin secretion via insulin-signalling pathways [29]. This finding concurs with a previous study (2004) conducted on participants with T2D who were given doses of the French Maritime pine bark (Pcynogenol^®^) ranging from 50 to 300 mg on separate occasions and reported significant improvements in fasting and postprandial glucose, and HbA1c without any statistically significant changes in insulin levels, thus excluding the possibility of insulin secretion enhancement as one of the mechanistic actions of pine bark in improving glycaemia [25]. Another 2020 study concluded that, depending on the composition of plant extracts and their relative bioactivity, some extracts were only able to demonstrate reductions in postprandial glucose without improvements in postprandial insulin levels in healthy adults, whilst others only influenced changes in postprandial insulin [34]. Nevertheless, there was an indication that postprandial blood glucose was reduced with 400 mg of the pine bark extract without the need to increase insulin secretion for the enhanced disposal of glucose, which may be protective against beta-cell dysfunction [35]. The finding is in contrast to an animal study (2014) that demonstrated reductions in HbA1c, insulin, and glucagon levels when pine bark extract (Enzogenol^®^) was administered to a diabetic mice model [36]. A larger variability in insulin levels was also observed within treatments in the current study, leading to the inability to detect potential statistical differences between the treatments. For future work, the measurement of *C*-peptide levels instead of insulin would provide more accurate readings of true portal insulin secretion and eliminate variability in the pulsative nature and hepatic clearance of insulin [37], in order to ascertain if indeed postprandial insulin secretion was not impacted by the co-administration of the pine bark extract with sucrose.

Postprandial glucose curve shapes deriving from the oral glucose tolerance test (OGTT) have been shown to be useful indicators of future T2D risk even in healthy individuals [38,39]. A monophasic glucose curve shape is defined by having only one peak in the glucose curve, and is associated with heightened T2D risk [40,41]. In contrast, both the biphasic and triphasic glucose curves shapes (complex) are defined as having at least two or more peaks, respectively, and are associated with better glycaemic outcomes and lower T2D risk [40,41]. A persistence in monophasic glucose curve shape and the shift from biphasic to monophasic glucose curve shape was associated with an increased risk of impaired glucose metabolism [42]. Interestingly, the current study showed that 86.8% of the participants exhibited monophasic glucose curve shapes with the control, 83.8% with 50 mg of the pine bark extract, and a lower 66.7% with 400 mg of the extract. Biphasic glucose curve shapes were observed in 13.2% of the participants with the control, 13.5% with 50 mg of the pine bark extract, and 30.8% with 400 mg of the extract. There was also 2.7% of the participants exhibiting triphasic glucose curve shapes with 50 mg of the pine bark extract and 2.6% with 400 mg of the extract. This indicates that the New Zealand pine bark, particularly with a higher dose (400 mg), was able to improve glucose curve shapes in healthy individuals in an acute manner.

Compared with our previous study on the New Zealand pine bark with glucose consumption in an OGTT [7], 400 mg of the pine bark extract was able to significantly reduce iAUCglucose compared to the control with the glucose solution (235.7 ± 16.5 vs. 299.5 ± 26.9 mmol/L·min, 21.3% reduction, and *p* = 0.016). The current study demonstrated that the pine bark extract was also able to significantly reduce iAUCglucose compared to the control with a sucrose solution (211.8 ± 13.9 vs. 247.5 ± 14.0 mmol/L·min, 14.4% reduction, and *p* = 0.037). This suggests that the New Zealand pine bark works via multiple pathways to regulate postprandial blood glucose, as it was shown in both studies that the pine bark extract was able to attenuate and reduce overall postprandial glucose via its inhibitory actions on the digestion and absorption of sucrose and glucose, two common carbohydrate sources in the diet. Nonetheless, further work is required to determine the exact mechanistic actions of the pine bark extract to determine why it was able to reduce glucose absorption to a greater extent than for sucrose. One explanation may be that as sucrose breaks down into glucose and fructose for absorption, the pine bark compounds might be active at the site of action for both the inhibition of sucrose digestion and glucose absorption in the small intestine, thus reducing its maximal effects in inhibiting sucrose digestion. The pine bark extract might also be potentially active on the fructose-specific transporter (glucose transporter 5, GLUT5) for fructose uptake during sucrose digestion [43], thus further limiting its enzymatic inhibitory capacity on sucrase. Another explanation might be that the structural inhibition of the pine bark polyphenols, predominantly high in proanthocyanidins, are more specific to the structure of glucose transporters rather than the sucrase enzyme, thus leading to a more fitting inhibition and greater improved glycaemic responses with glucose than with sucrose consumption. More recent reviews suggest that plant extracts high in proanthocyanidins could have differential inhibitory effects on carbohydrate digestion enzymes depending on their respective phenolic compositions, complexity, and polymerisation [19,44], such that proanthocyanidin oligomers may be preferred over longer-chain polymers as better alpha-glucosidase inhibitors for diabetes prevention and management [45]. Additionally, dependent on the structure, active sites, and composition of phenolic compounds present in plant extracts, the extracts could exhibit more efficacious inhibitory intensities towards specific glucose transporters (e.g., sodium-glucose cotransporter (SGLT1) or glucose transporter 2 (GLUT2)), leading to reduced glucose uptake across the intestinal brush border membrane and into the bloodstream [43,46].

One study done by Wang and co-workers (2018) concluded that the actions of mulberry leaf plant extract on maltose, maltodextrin, and sucrose produced lower postprandial glucose levels in healthy participants than when mulberry leaf extract was consumed with glucose alone [21]. However, the study did not compare intervention treatments with their respective carbohydrate sources, as also noted by the authors, such as comparing sucrose combined with Reducose^®^ and sucrose (control) only, rather than with glucose, as carbohydrates may raise blood glucose by different magnitudes (e.g., dietary glucose raises blood glucose and insulin levels to a greater extent than sucrose) [47]. Therefore, it remains inconclusive as to whether mulberry leaf extract was able to inhibit sucrose digestion better than glucose absorption. A merit of the current study was that sucrose, in combination with the pine bark extract, was compared to a control (sucrose only), in order to accurately quantify the glycaemic improvements.

Although it is generally accepted that a 20% or more reduction in postprandial iAUCglucose would be considered as clinically significant for the management of glycaemia [48,49], a 14.4% reduction in iAUCglucose with 400 mg of pine bark extract as demonstrated in the present study would still provide a useful means to optimise glycaemic control, if taken on a consistent basis. This is especially so when hyperglycaemic excursions after a meal and daily fluctuating blood glucose contribute to increased oxidative stress and endothelial dysfunction, leading to increased T2D and cardiovascular risk [50]. Therefore, even a small but consistent improvement in postprandial glycaemic responses may be beneficial for healthy individuals without diabetes [51]. The effect could potentially be more pronounced in individuals with prediabetes with elevated fasting glucose or impaired glucose tolerance, or T2D. Further studies should investigate the longer-term effects of the New Zealand pine bark on glycaemic measures such as HbA1c, glycaemic variability, beta-cell function, and cardiovascular risk factors such as blood lipids and blood pressure, in order to determine the overall clinical significance of the New Zealand pine bark on postprandial glycaemia.

Further merits of this study include the use of a crossover design which minimised interindividual variation amongst treatments, and a larger sample size to determine the efficacy of the New Zealand pine bark on postprandial glycaemic responses in humans.

Nevertheless, this study is not without limitations. Although this study was conducted using sucrose solution to provide insights into the potential inhibitory effect of the pine bark extract on sucrose digestion by sucrase enzyme, we did not quantify the degree of undigested sucrose such as through hydrogen breath tests to ascertain that sucrose has indeed not been digested, resulting in reduced postprandial blood glucose levels [34,52].

The clinical impacts of the New Zealand pine bark on glycaemic responses may be attributed to its inhibitory effects on digestive enzymes, such as sucrase. Notwithstanding, further mechanisms might have also acted in concert, by the phenolic compounds improving glycaemia, such as the enhancement of incretin levels that stimulate insulin secretion towards increased glucose uptake, the inhibition of glucose transport and uptake [53], the activation of hepatic glucokinase, glycogen synthase activity, and AMP-activated protein kinase (AMPK), and the suppression of glucose-6-phosphatase (G-6-Pase) and phosphoenolpyruvate carboxykinase (PEPCK) expressions leading to a decrease in gluconeogenic enzyme activity [36], which have been previously demonstrated with pine bark extracts using in vitro and rat models. Further work is warranted to determine other mechanistic actions of the pine bark extract that might be involved in reducing glucose absorption to support the improvement in glycaemic responses seen in the present study.

Future clinical studies should therefore examine other physiological parameters, such as incretin glucose-dependent insulinotropic polypeptide (GIP) and glucagon-like peptide-1 (GLP-1) levels to ascertain their impact on glycaemic responses upon pine bark extract administration. The improvements in incretin levels have recently been demonstrated in other plant extracts with similar inhibitory functions to the New Zealand pine bark [54,55]. The New Zealand pine bark may also be examined in composite meals to determine its effectiveness in regulating postprandial glycaemia after meals, which are more representative of what is normally consumed. Longer-term consumption of the pine bark extract should be tested to examine its efficacy and safety in regulating glycaemic responses. Further research should also examine the impact of the New Zealand pine bark in a cohort of individuals with prediabetes or T2D to determine its effectiveness in improving glycaemic control in this population group.

## 5. Conclusions

The current study demonstrated that the New Zealand pine bark leads to better postprandial glycaemic responses in participants fed a sucrose solution. The results suggest an inhibitory effect of the New Zealand pine bark on sucrose digestion and uptake, potentially via sucrase enzyme inhibition. This adds to the current knowledge of plant-based extracts such as pine bark extract that may potentially be useful adjuncts to the diet for the healthy maintenance of postprandial glycaemia.

## Figures and Tables

**Table 1:** Baseline characteristics of participants (n = 40) ± SEM.

Characteristics	Male (n = 12)	Female (n = 28)	Mean (n = 40)
Age (year)	33.8 ± 2.2	30.1 ± 1.6	30.1 ± 1.3
Body Mass Index (BMI, kg/m^2^)	23.8 ± 0.8	23.2 ± 0.6	23.4 ± 0.5
Waist circumference (cm)	83.0 ± 2.2	74.5 ± 1.7	77.0 ± 1.5
Hip circumference (cm)	99.5 ± 2.3	98.7 ± 1.5	98.9 ± 1.2
Systolic blood pressure (mmHg)	113 ± 3.3	106 ± 1.7	108 ± 1.6
Diastolic blood pressure (mmHg)	74 ± 2.0	68 ± 1.1	70 ± 1.0
Fasting glucose (mmol/L)	4.5 ± 0.2	4.7 ± 0.1	4.7 ± 0.1
Glycated haemoglobin A1c (HbA1c, mmol/mol)	32.8 ± 1.0	32.3 ± 0.7	32.5 ± 0.6
Total cholesterol (TC, mmol/L)	4.7 ± 0.3	4.6 ± 0.2	4.7 ± 0.1
Triglyceride (TG, mmol/L)	1.2 ± 0.1	1.3 ± 0.1	1.3 ± 0.1
High density lipoprotein (HDL, mmol/L)	1.5 ± 0.1	1.7 ± 0.1	1.6 ± 0.1
Low density lipoprotein (LDL, mmol/L)	2.7 ± 0.2	2.3 ± 0.2	2.4 ± 0.1
Non-high-density lipoprotein (non-HDL, mmol/L)	3.2 ± 0.3	2.9 ± 0.2	3.0 ± 0.1
TC/HDL ratio	3.1 ± 0.2	2.9 ± 0.2	3.0 ± 0.1

**Table 2:** Results of incremental area under the curve (iAUC) of postprandial glucose (mmol/L⋅min) (± SEM) at 30, 60, 90, and 120 min (which is also the iAUC mean), 1 h glucose (mmol/L) (± SEM), glucose peak value (mmol/L) (± SEM), and glucose peak time (min) (± SEM) of participants (n = 40) in each intervention (50 and 400 mg of pine bark extract compared to control). * Statistically significant difference detected compared to control (*p* < 0.05).

	Control (Only Sucrose Solution)	50 mg Pine Bark Extract	400 mg Pine Bark Extract
iAUC 30	62.2 ± 3.4	58.1 ± 3.5	62.3 ± 3.4
iAUC 60	163.4 ± 8.1	152.7 ± 8.1	147.5 ± 8.0
iAUC 90	223.1 ± 12.0	203.1 ± 12.1	192.3 ± 11.9 *
iAUC 120	247.5 ± 14.0	220.8 ± 14.2	211.8 ± 13.9 *
1 h glucose	7.5 ± 0.2	7.3 ± 0.2	7.0 ± 0.2
Glucose peak	9.1 ± 0.2	8.6 ± 0.2 *	8.7 ± 0.2
Glucose peak time	35.8 ± 1.8	36.7 ± 1.9	31.3 ± 1.8

**Table 3:** Mean postprandial glucose (mmol/L) (± SEM) of participants (n = 40) with time in each intervention (50 and 400 mg of pine bark extract compared to control). * Statistically significant difference detected compared to control (*p* < 0.05).

Time (min)	Control (Only Sucrose Solution)	50 mg Pine Bark Extract	400 mg Pine Bark Extract
0	4.9 ± 0.1	4.8 ± 0.1	4.9 ± 0.1
15	7.1 ± 0.2	7.0 ± 0.2	7.3 ± 0.2
30	8.7 ± 0.2	8.2 ± 0.2	8.4 ± 0.2
45	8.4 ± 0.2	8.1 ± 0.2	7.8 ± 0.2 *
60	7.5 ± 0.2	7.3 ± 0.2	7.0 ± 0.2
90	6.1 ± 0.2	5.7 ± 0.2 *	5.7 ± 0.2
120	4.8 ± 0.2	4.6 ± 0.2	4.9 ± 0.2

**Table 4:** Results of incremental area under the curve (iAUC) of postprandial insulin (mU/L⋅min) (± SEM) at 30, 60, 90, and 120 min (which is also the iAUC mean), 1 h insulin (mU/L) (± SEM), insulin peak value (mmol/L) (± SEM), and insulin peak time (min) (± SEM) of participants (n = 40) in each intervention (50 and 400 mg of pine bark extract compared to control).

	Control (Only Sucrose Solution)	50 mg Pine Bark Extract	400 mg Pine Bark Extract
iAUC 30	1094.11 ± 122.67	939.31 ± 124.86	1001.49 ± 123.54
iAUC 60	2787.52 ± 288.11	2797.96 ± 290.89	2547.35 ± 287.53
iAUC 90	4021.70 ± 413.63	4059.75 ± 417.78	3629.88 ± 412.78
iAUC 120	4601.86 ± 499.39	4873.98 ± 504.54	4231.87 ± 498.35
1 h insulin	60.26 ± 6.76	67.77 ± 6.70	50.65 ± 6.69
Insulin peak	81.74 ± 7.59	77.99 ± 7.63	76.19 ± 7.54
Insulin peak time	37.2 ± 2.8	35.6 ± 2.8	37.8 ± 2.8

**Table 5:** Mean postprandial insulin (mU/L) (± SEM) of participants (n = 40) with time in each intervention (50 and 400 mg of pine bark extract compared to control).

Time (min)	Control (Only Sucrose Solution)	50 mg Pine Bark Extract	400 mg Pine Bark Extract
0	8.12 ± 0.79	7.97 ± 0.80	8.41 ± 0.79
15	53.42 ± 6.02	54.03 ± 6.15	55.10 ± 6.01
30	74.06 ± 7.59	64.29 ± 7.73	67.54 ± 7.63
45	66.34 ± 6.76	66.35 ± 6.80	61.32 ± 6.72
60	60.26 ± 6.76	67.77 ± 6.70	50.66 ± 6.69
90	38.86 ± 5.50	40.79 ± 5.61	34.28 ± 5.45
120	18.66 ± 3.57	18.08 ± 3.47	21.39 ± 3.36

## Data Availability

The original contributions presented in this study are included in the article. Further inquiries can be directed to the corresponding author.

## Abbreviations

BMI	Body Mass Index
DBP	Diastolic blood pressure
EAT-26	The Eating Attitudes Testing (questionnaire)
FBG	Fasting blood glucose
HbA1c	Glycated haemoglobin A1c
HDL	High-density lipoprotein cholesterol
LDL	Low-density lipoprotein cholesterol
iAUC	Incremental area under the curve
iAUCglucose	Incremental area under the curve of postprandial glucose
IPAQ	International Physical Activity Questionnaire
OGTT	Oral glucose tolerance test
SBP	Systolic blood pressure
TC	Total cholesterol
TG	Triglyceride
1hPG	1 h postprandial glucose
2hPG	2 h postprandial glucose
